# Sitagliptin ameliorates busulfan-induced pulmonary and testicular injury in rats through antioxidant, anti-inflammatory, antifibrotic, and antiapoptotic effects

**DOI:** 10.1038/s41598-023-36829-3

**Published:** 2023-06-16

**Authors:** Eman A. Ali, Sara G. Tayel, Mona A. Abbas

**Affiliations:** 1 Clinical Pharmacology Department, Faculty of Medicine, Menoufia University, Menoufia, Egypt; 2 Anatomy and Embryology Department, Faculty of Medicine, Menoufia University, Menoufia, Egypt; 3 Medical Biochemistry and Molecular Biology Department, Faculty of Medicine, Menoufia University, Menoufia, Egypt

**Keywords:** Biochemistry, Clinical pharmacology

## Abstract

Busulfan (BUS) is an anticancer agent with serious adverse effects on various body organs, including the lung and testis. Sitagliptin was proven to have antioxidant, anti-inflammatory, antifibrotic, and antiapoptotic effects. This study aims to evaluate whether sitagliptin, a DPP4I, ameliorates BUS-induced pulmonary and testicular injury in rats. Male Wistar rats were split into control, sitagliptin (10 mg/kg), BUS (30 mg/kg), and sitagliptin + BUS groups. Weight change, lung and testis indices, serum testosterone, sperm parameters, markers of oxidative stress [malondialdehyde (MDA) and reduced glutathione (GSH)], inflammation [tumor necrosis factor-alpha (TNF-α)], and relative expression of sirtuin1 (*SIRT1*) and forkhead box protein type O1 (*FOXO1*) genes were estimated. Histopathological examination of lung and testicular tissues was done to detect architectural changes [Hematoxylin & Eosin (H&E)], fibrosis (Masson’s trichrome), and apoptosis (caspase-3). Sitagliptin treatment reduced body weight loss, lung index, lung and testis MDA, serum TNF-α and sperm abnormal morphology, and increased testis index, lung and testis GSH, serum testosterone, sperm count, viability and motility. *SIRT1/FOXO1* balance was restored. Also, sitagliptin attenuated fibrosis and apoptosis in lung and testicular tissues via reducing collagen deposition and caspase-3 expression. Accordingly, sitagliptin ameliorated BUS-induced pulmonary and testicular damage in rats via attenuating oxidative stress, inflammation, fibrosis, and apoptosis.

## Introduction

Busulfan (BUS) is an alkylating anticancer agent for treating leukemia, lymphoma, and ovarian cancer. Moreover, BUS is used in cancer patients prior to transplanting bone marrow^1^. However, it has deleterious effects on many body organs, such as the lungs^2^ and testes^3^.

According to reports, BUS is considered the first cytotoxic agent linked to pulmonary toxicity^4^ in up to 8% of BUS-treated patients resulting in acute pulmonary injury, chronic interstitial fibrosis, and hemorrhagic lesions in the alveoli^2^. BUS causes alveolar damage by exerting a direct toxic effect on the respiratory epithelium, especially type II pneumocytes leading to interstitial edema and pulmonary fibrosis^2^.

In addition, spermatogenesis can be markedly influenced by chemotherapeutic agents due to the high division rate of the testes leading to sterility^5^. When taken for long periods, BUS leads to testicular toxicity, including disruption of spermatogenesis, germ cell apoptosis, oligo-azoospermia, and sperm abnormality^3,5^. The link between lung and testicular toxicity could be attributed to their high vulnerability to damage from BUS^5,6^. Also, both organs are postulated to share the same mechanism of BUS toxicity^7,8^. Accordingly, the effect of BUS on these organs reflects the need for careful monitoring of patients treated with BUS for side effects.

The mechanism of BUS toxicity remains not elucidated. However, it is thought that it could be due to interrupting deoxyribonucleic acid (DNA) replication by binding to DNA single strand as well as inhibiting ribonucleic acid (RNA) transcription process, leading to activation of oxidative stress, inflammatory cytokines including tumor necrosis factor-α (TNF-α), fibrosis, and apoptosis^1,7,9^.

Sirtuin1 (*SIRT1*) is a nicotinamide adenine dinucleotide- (NAD+) dependent class III histone deacetylase, which catalyzes many protein substrates to perform its functions and is broadly expressed in many tissues. *SIRT1* handles numerous pathways, for example, oxidative stress, inflammation, fibrosis, apoptosis, and autophagy. *SIRT1* regulates many proteins, including forkhead box protein type O1 (*FOXO1*)^10,11^. The deacetylation of *FOXO1* by *SIRT1* reduces its transcriptional activity^12^. *SIRT1/FOXO1* interaction is crucial in modulating oxidative stress and apoptosis^12,13^. The impact of *SIRT1/FOXO1* interaction in BUS-induced pulmonary or testicular injury has not been fully demonstrated. This study tends to highlight the modulation of this interaction as a future therapeutic target.

Dipeptidyl peptidase 4 (DPP4) is an ectopeptidase bound to the cell membrane and expressed in many tissues. It has been involved in many pathways, for example, oxidative stress, inflammation, apoptosis, signal transduction, immune response regulation, and glucose metabolism^14,15^. Lungs are considered the second highest organ for DPP4 expression. Its overexpression has been linked to several respiratory system disorders, like lung fibrosis, obstructive lung diseases, bronchial asthma, and lung cancer^16^. Additionally, DPP4 is expressed in the testicular tissue^17^, making it a promising therapeutic target for treating pulmonary and testicular toxicity caused by BUS.

Sitagliptin is a dipeptidyl peptidase 4 inhibitor (DPP4I)^18^. Previous studies have proved that DPP4Is had various effects, including antioxidant^19^, anti-inflammatory^16^, antifibrotic, and antiapoptotic features^20,21^. According to our knowledge, the influence of sitagliptin on BUS-induced pulmonary or testicular injury has not been studied before. The current research aims to clarify the potential protective effect of sitagliptin in ameliorating BUS-induced pulmonary and testicular injury in rats.

## Materials and methods

### Animals

In total, 40 12-week-old male Wistar rats (200–250 g) were obtained and adapted for one week before the beginning of the research in an appropriate environment with unrestricted accessibility to water and diet. The experiment followed the ARRIVE guidelines and the Guide for the Care and Use of Laboratory Animals approved by the National Institute of Health. The study was approved by the Ethical Committee of the Faculty of Medicine, Menoufia University, Menoufia, Egypt (Permit Number: 10/2022 BIO6-4). All methods were performed in accordance with the relevant guidelines and regulations.

### Drugs and experimental model

After determining the rats’ baseline body weight, they were equally (10 rats per group) allocated into four groups. Control: rats were injected with saline 0.9% orally (*P.O.*) for 4 weeks starting a week before phosphate-buffered saline (PBS) intraperitoneal (*i.p.*) injection. Sitagliptin: rats were injected with sitagliptin (*P.O.*) for 4 weeks starting a week before injecting PBS (*i.p.*). BUS: BUS was brought from Sigma-Aldrich Chemical Co. (St. Louis, MO, USA (Cat. No. B2635)) and injected into rats *i.p.* starting from the second week at a dose of 30 mg/kg. The total dose was divided into two equal doses (15 mg/kg each) and given over two weeks with a one-week interval (on days 8 and 15). BUS was dissolved in 2.5 ml of PBS/dose to yield 0.5 ml/rat. The BUS dose used in the current study was sufficient to cause lung injury and seminiferous epithelium impairment with the least animal mortality when given as an i.p. injection, according to previous studies^7,22,23^. In addition, the i.p. route guarantees systemic exposure of animals to the drug and mimics the intravenous route, which is the exposure route in humans^24,25^. Sitagliptin + BUS: rats were injected with sitagliptin (10 mg/kg orally^26^) and BUS (as in the BUS group). Sitagliptin was given for four weeks, starting a week before injecting BUS. Sitagliptin dose was dissolved in 5 ml saline 0.9% to yield 1 ml/rat. Sitagliptin (Januvia) was brought commercially from Merck Sharp and Dohme (MSD) Co., Whitehouse Station, New Jersey, USA. The selected dose of sitagliptin was confirmed to have antioxidant, anti-inflammatory, antifibrotic, and antiapoptotic effects against chemotherapy-induced cardiac and testicular injury^21,27^ and experimentally induced nonalcoholic fatty liver disease^28^.

On the last day of the experiment, rats from all groups were weighed to estimate final body weight (FBWt). After that, blood samples were gathered, and then all animals were sacrificed by decapitation to obtain tissue samples for biochemical and histopathological evaluation.

### Collection of blood samples

Ketamine (75 mg/kg) and xylazine (5 mg/kg) i.p. anesthesia was used to obtain a 2 ml blood sample from the retro-orbital plexus of each rat. The blood samples were gathered in capillary tubes to which heparin was added to determine serum TNF-α and testosterone levels^29^. At room temperature, the samples were kept until clotting, followed by centrifugation at 2000 rpm for 15 min. After that, the gathered samples were preserved at – 80 °C. Serum testosterone and TNF-α levels were estimated by ELISA kits provided by Sigma-Aldrich Chemical Co. (St. Louis, MO, USA) (Cat. No. SE120089) and BD Biosciences. Inc., San Diego, CA, USA (Cat. No. 558535), respectively.

### Collection of tissue samples, assessing sperm parameters, and determination of lung and testis indices

Rats were decapitated, testes were extracted, the thoracic cavity was opened, and lungs were collected. After extraction, the right lung and the right testis of each rat were divided into two halves; one half was used to assess malondialdehyde (MDA) and reduced glutathione (GSH) levels, and the other half was employed in a real-time polymerase chain reaction (RT-PCR). Left lungs and testes were fixed in saline for histopathological evaluation. To obtain seminal fluid for measuring sperm parameters, the epididymis’ caudal portion of each rat was crumbled with scissors and put into 5 mL of saline^30^. The seminal fluid obtained from cauda epididymis was placed on a slide. The motile spermatozoa/unit area was counted and identified as sperm motility and represented as motility/unit area. Hemocytometer was used to count sperms. Sperm count was expressed as millions/ml of suspension. A smear of spermatozoa was prepared on slides and stained with an eosin-nigrosine stain to assess sperm viability, according to Raji and Bolarinwa^31^. The aniline blue staining technique was applied to evaluate sperm morphology, and percentages were used to represent abnormal morphologies. Sperms’ cytoplasmic residual was regarded as an aberrant morphology^32^.

For each rat, both lungs were weighed for determination of the lung index (lung weight (g)/ FBWt. (g)^33^ × 100). Also, the testes of each rat were weighed for determination of the testis index (testes weight (g)/ FBWt. (g) × 100)^34^.

### Measurement of lung and testis MDA and GSH levels

The lungs and testes samples were perfused in PBS solution (pH 7.4, with heparin added) to eliminate red blood cells (RBCs) or clots, then homogenized in cold buffer (pH 7.5, 50 mM potassium phosphate and 1 mM EDTA) using a glass tissue homogenizer. The homogenate was subjected to centrifugation at 4000 rpm for 20 min, and the supernatant was kept at – 80 °C to measure MDA^35^ (MD 25 29; Biodiagnostic, Dokki, Giza, Egypt) and GSH^36^ (GR 25 11; Biodiagnostic, Dokki, Giza, Egypt) levels.

### Measurement of *FOXO* and *SIRT1* genes’ relative expression by RT-PCR

**Figure 1:**
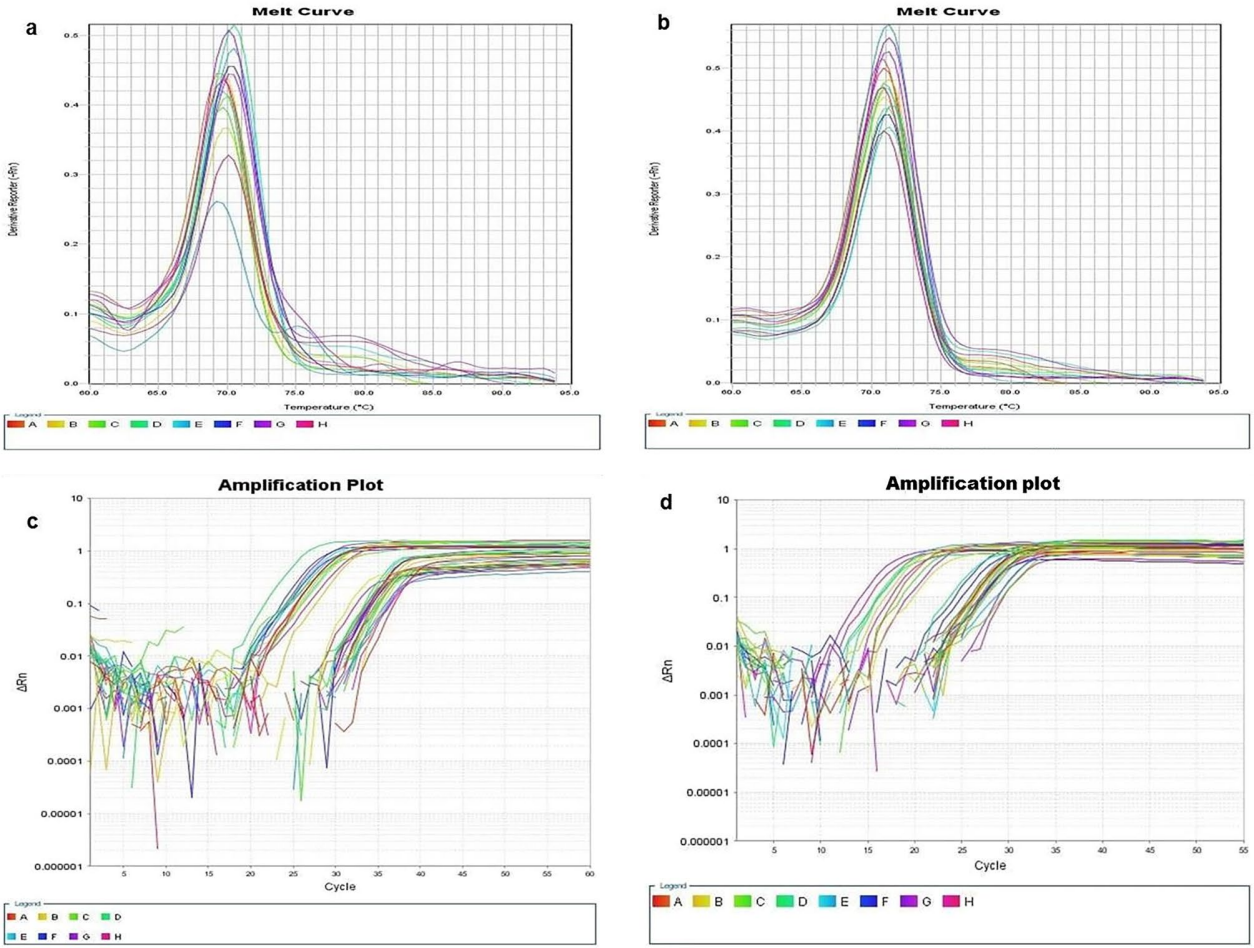
(**a**) Melting curve of *SIRT1* and *FOXO1* expression in lung. (**b**) Melting curve of *SIRT1* and *FOXO1* expression in testis. (**c**) Amplification plot of *SIRT1* and *FOXO1* genes in lung. (**d**) Amplification plot of *SIRT1* and *FOXO1* genes in testis.

The lung and testicular tissues were kept at 4 °C overnight in RNA*Later* Solution (AM7020; Thermo Fisher Scientific, Waltham, MA, USA), then at − 80 °C until processing. Before processing, the samples were homogenized with TissueLyser LT (QIAGEN, Hilden, Germany). Total cellular RNA was extracted using the miRNeasy Mini Kit (217084; QIAGEN, Germany) per the manufacturer’s directions. The concentration of the RNA excerpt was then assessed using a NanoDrop spectrophotometer (ThermoScientific, Waltham, MA, USA). The extracted RNA was reverse-transcribed using the RevertAid First Strand cDNA Synthesis Kit (K1622; ThermoScientific, Waltham, MA, USA), and the applied biosystems 2720 thermal cycler was set to 25 °C for 5 min, then 42 °C for 60 min and terminated at 70 °C for 5 min. The RT product was kept at − 20 °C for RT-PCR. Finally, cDNA amplification was done using SYBR green-based quantitative real-time PCR by maxima SYBR green qPCR master mix Kit (K0221; ThermoFisher Scientific, Waltham, MA, USA). The total volume of 20 μl was made up of 10 μl of SYBR green, 7 μl of nuclease-free water, 1 μl of template cDNA, and 1 μl of each forward and reverse primer. The primers were supplied by QIAGEN, Germany. Table 1 lists the primer pairs employed. β-Actin was used as a housekeeping gene. The Primer-BLAST software was used to confirm the primer sequence. The cycling program included initial denaturation for 10 min at 95 °C followed by 45 cycles for 15 s at 95 °C, annealing for 30 s at 60 °C and extension for 30 s at 72 °C. The 7500 ABI PRISM instrument software version 2.0.1 (Applied Biosystems, USA) was used for data analysis, as in Fig. 1. The 2^−ΔΔCT^ equation was employed to compute gene expression fold changes.

**Table 1:** Primers designed for amplifying sirtuin1 *(SIRT1)*, forkhead box protein type O1 (*FOXO1),* and *β-actin* genes*.*

SIRT1	Forward	5′-AGC TGG GGT TTC TGT TTC CTG TGG-3′
	Reverse	5′-CGA ACA TGG CTT GAG GAT CTG GGA-3′
FOXO1	Forward	5′-GAT AAG GGC GAC AGC AAC AG-3′
	Reverse	5′-TGA GCA TCC ACC AAG AAC TT-3′
β-Actin	Forward	5′-CAC ACC CGC CAC CAG TTCG-3′
	Reverse	5′-ACC CAT TCC CAC CAT CAC ACC-3′

### Histopathological examination

The left lungs and testes were kept in 10% formalin solution, cleared, and entrenched in five-micrometer sections. Then, sections were stained with H&E to point out the histological changes^37^. Also, Masson’s trichrome stain was used to distinguish collagen fiber deposition^38^. Mouse monoclonal primary antibody to caspase-3 (A17900; Ab-7, Mouse Mab. MS.) was considered the primary monoclonal antibody. Brownish cytoplasm and/or nuclei were indicative of its reaction.

For caspase-3 immunostaining, deparaffinized 5 µm lung and testis sections were rehydrated in declining alcohol grades and then embedded in 3% hydrogen peroxide (H_2_O_2_) for 10 min to impede endogenous peroxidase. After that, sections were submerged in an antigen retrieval solution containing 10 mmol/l sodium citrate buffer (pH 6.0). The binding of nonspecific proteins was avoided by adding a blocking solution (PBS and 10% normal goat serum). The primary antibody (caspase-3 ab49822; Abcam, Trumpington, Cambridge, UK) chosen dilution to incubate with the tissue sections was 1:500, after which the secondary biotinylated antibodies (21538 M; goat anti-mouse IgG, Sigma-Aldrich, St. Louis, USA) were applied for about 20 min. The sections were incubated with a preformed streptavidin peroxidase complex. The sections were incubated with 3,3′-diaminobenzidine tetrahydrochloride (DAB D5905; Sigma-Aldrich, St. Louis, MO, USA) to identify the secondary antibody binding. Lastly, PBS solution was used to dip slices to be counterstained with H to be examined using a light microscope^39^.

### Morphometric analysis

For quantitative evaluation of the number of cells with a positive reaction to caspase-3 and area percentage of collagen fiber deposition, Image J 1.47v software (National Institutes of Health, USA) was used. Five nonoverlapping fields per section were chosen arbitrarily, and the calculated values were compared and statistically analyzed.

### Statistical analysis

To detect the normality of data, the Shapiro–Wilk test was used. One-way ANOVA was used to analyze the parametric data, and then the Tukey test was applied as a post hoc test. Mean and standard deviation (SD) represented the data. Statistical tests were two-tailed, and results were statistically significant at *P* < 0.05. GraphPad Prism software version 7 (GraphPad Software Inc., San Diego, CA, USA) was used to analyze the collected data.

## Results

### The effect of sitagliptin on body weight change, lung and testis indices, and oxidative stress parameters

**Figure 2:**
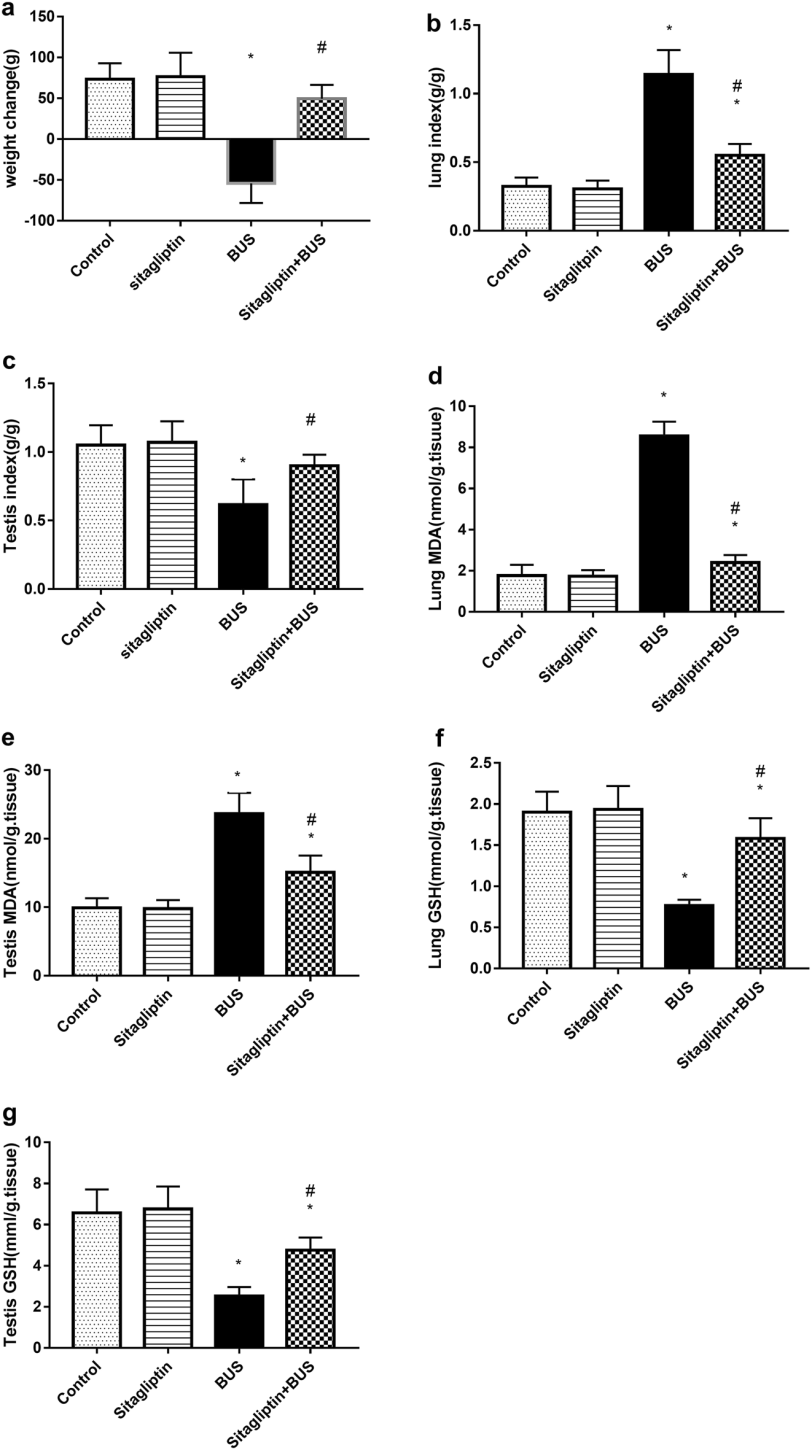
The effect of sitagliptin and BUS on (**a**) body weight change, (**b**) lung index, (**c**) testis index, MDA levels in (**d**) lung and (**e**) testis, and GSH levels in (**f**) lung and (**g**) testis. Bars represent mean ± SD (n = 10). One-way ANOVA and the Tukey post hoc test were used to evaluate the data. *P < 0.05 compared to the control group; ^#^*P* < 0.05 compared to the BUS group.

As shown in Fig. 2a, the BUS group showed marked body weight loss compared to the control group (*P* < 0.05). The sitagliptin-treated group exhibited a marked increase in weight gain compared to the BUS group (*P* < 0.05). The reduction in body weight loss in the group treated by sitagliptin was nonsignificant (*P* > 0.05) compared to the control group. In Fig. 2b, BUS markedly increased lung index (*P* < 0.05) compared to the control group, and sitagliptin treatment significantly reduced lung index compared to the BUS group (*P* < 0.05). The lung index in the sitagliptin-treated group was significantly higher than that in the control group (*P* < 0.05). Testis index showed a notable reduction in the BUS group (*P* < 0.05) compared to the control group. Treatment with sitagliptin significantly (*P* < 0.05) increased the testis index compared to the BUS group. The reduction in testis index was nonsignificant (*P* > 0.05) when compared to the control group (Fig. 2c).

Also, Fig. 2d revealed that lung MDA levels in the BUS group were significantly higher (*P* < 0.05) than that in the control group. Sitagliptin treatment showed a notable decline in MDA in lung tissue (*P* < 0.05) compared to the BUS group. Nevertheless, treatment with sitagliptin failed to reach the normal levels of MDA (*P* < 0.05) compared to the control group. Similarly**,** testis MDA levels in the BUS group were notably elevated (*P* < 0.05) compared to the control group. The sitagliptin-treated group declined testis MDA (*P* < 0.05) compared to the BUS group. However, treatment with sitagliptin could not normalize (*P* < 0.05) testis MDA level compared to the control group (Fig. 2e).

BUS induced a significant reduction in lung GSH level (*P* < 0.05) compared to the control group. Sitagliptin treatment reversed the reduction in lung GSH compared to the BUS group (*P* < 0.05). Nevertheless, lung GSH level in the sitagliptin-treated group was lower than the normal GSH levels of the control group (*P* < 0.05) (Fig. 2f). In a similar fashion, the BUS group exhibited a marked decline in testis GSH level (*P* < 0.05) compared to control rats. Even though sitagliptin treatment showed notable elevation in testis GSH when compared to the BUS group, the GSH level was still lower than the control group level (*P* < 0.05) (Fig. 2g).

### The effect of sitagliptin on serum TNF-α level, testosterone level, and sperm parameters

**Figure 3:**
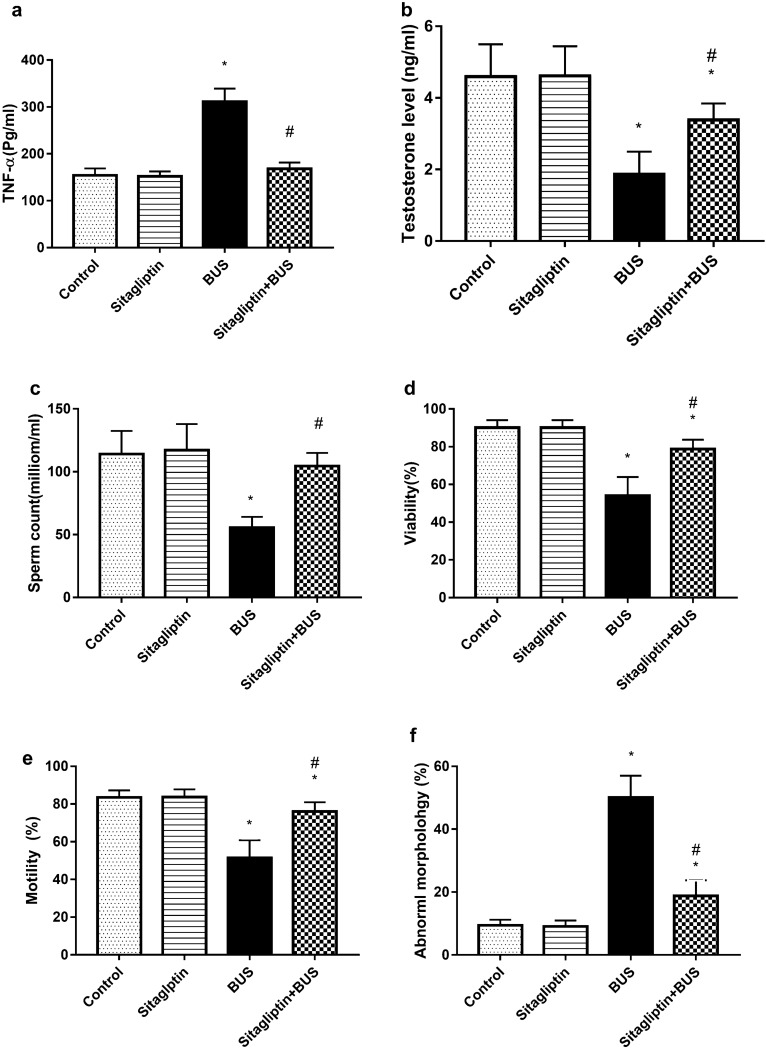
The effect of sitagliptin and BUS on (**a**) serum levels of tumor necrosis factor-α (TNF-α), (**b**) testosterone, (**c**) sperm count, (**d**) sperm viability and (**e**) motility, and (**f**) sperm abnormal morphology. Bars represent mean ± SD (n = 10). One-way ANOVA and the Tukey post hoc test were used to evaluate the data. *P < 0.05 compared to the control group; ^#^*P* < 0.05 compared to the BUS group.

Figure 3a demonstrates that the BUS group had a significantly higher level of TNF-α than the control group (*P* < 0.05). Sitagliptin treatment significantly reduced the TNF-α level (*P* < 0.05) compared to the BUS group and returned the TNF-α level to normal values (*P* > 0.05) of the control group.

Also, the BUS group showed a significant reduction in testosterone serum level and the sitagliptin-treated group had a marked increase in testosterone level compared to the BUS group (*P* < 0.05). However, the testosterone level was still lower than that in the control rats (Fig. 3b).

Regarding sperm parameters, BUS-induced marked reduction in sperm count (*P* < 0.05) as compared to the control group, however, sitagliptin treatment returned the sperm count to the control rats’ values (*P* > 0.05) (Fig. 3c). Sperm viability was significantly declined as a result of BUS administration when compared to that of control rats (*P* < 0.05). However, the sitagliptin-treated group had higher sperm viability than the BUS group but lower than the control group’s viability level (*P* < 0.05) (Fig. 3d). Similarly, animals treated with BUS showed a decreased level of sperm motility (*P* < 0.05). Treatment with sitagliptin increased motility compared to the BUS group (*P* < 0.05). However, sperm motility in the sitagliptin-treated group was lower than the control group levels (*P* < 0.05) (Fig. 3e). Compared to the control group, abnormal sperm morphology was noticeably increased after BUS treatment (*P* < 0.05). Sitagliptin-treated group attenuated abnormal morphology compared to the BUS group (*P* < 0.05) without attaining the normal levels of control rats (*P* < 0.05) (Fig. 3f).

### The effect of sitagliptin on *SIRT1* and *FOXO1* genes’ relative expression in the lung and testis

**Figure 4:**
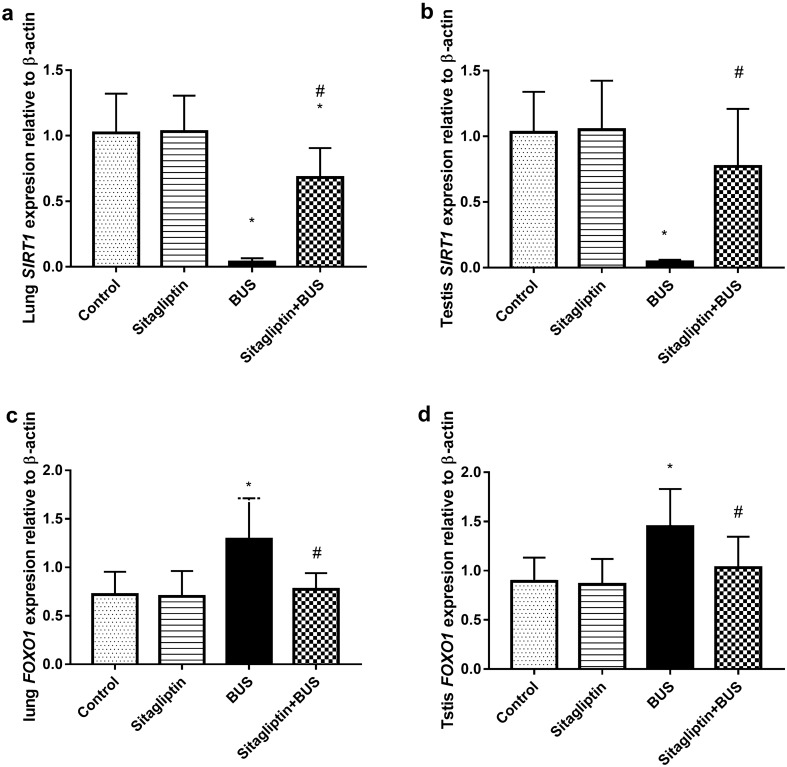
The effect of sitagliptin and BUS on *SIRT1* expression in (**a**) lung and (**b**) testis and *FOXO1* expression in (**c**) lung and (**d**) testis. Bars represent mean ± SD (n = 10). One-way ANOVA and the Tukey post hoc test were used to evaluate the data. *P < 0.05 compared to the control group; ^#^P < 0.05 compared to the BUS group.

As displayed in Fig. 4a, lung *SIRT1* expression in the BUS group showed significant attenuation (*P* < 0.05) compared to the control group. Treatment with sitagliptin markedly upregulated *SIRT1* expression (*P* < 0.05) compared to the BUS group. However, lung *SIRT1* expression in the sitagliptin-treated group was lower than that in the control group (*P* < 0.05). Additionally, BUS administration markedly reduced testis SIRT1 expression (*P* < 0.05) compared to the control group. Sitagliptin treatment resulted in lower testis *SIRT1* expression levels than the BUS group (*P* < 0.05). When compared to control rats, the sitagliptin-treated group showed no difference (*P* > 0.05) regarding the expression of testis *SIRT1* expression (Fig. 4b). BUS increased *FOXO1* expression in the lung (*P* < 0.05) compared to the control group. Lung *FOXO1* expression was markedly downregulated by sitagliptin treatment (*P* < 0.05) compared to the BUS. In addition, sitagliptin treatment reverted lung *FOXO1* expression to normal control levels (*P* > 0.05) (Fig. 4c). In a similar manner, BUS increased *FOXO1* expression in testis (*P* < 0.05) when compared to the control group. Sitagliptin treatment downregulated testis *FOXO1* expression compared to the BUS group (*P* < 0.05) and managed to restore testis *FOXO1* expression to normal levels of the control group (Fig. 4d).

### The effect of sitagliptin on H and E stain

**Figure 5:**
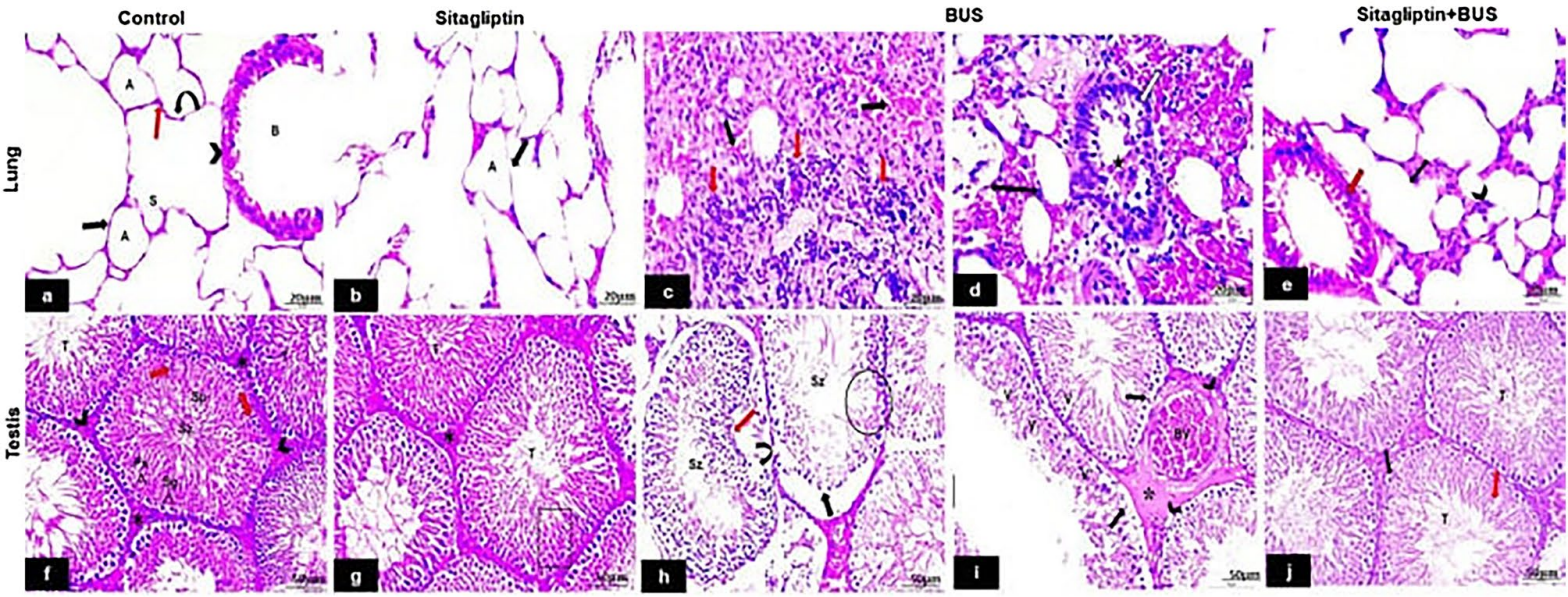
H&E staining of lung and testis tissues from different groups. Lung. (**a**) Control: exhibiting patent lung alveoli (A) lined with type I pneumocyte cells with flat nuclei (black arrow), type II pneumocyte cuboidal cells (red arrow) with fine interalveolar septa (curved arrow) and alveolar sacs (S). Bronchus (B) lined by columnar ciliated epithelium (arrowhead). (**b**) Sitagliptin group: showing normal lung architecture with normal patent alveoli (A) and fine interalveolar septa (arrow). (**c**) BUS group: showing disordered construction of the lung with marked inflammatory cellular invasion (red arrows) and leaking out of red blood cells (RBCs) (black arrows). (**d**) Thickened interalveolar septa (double-headed arrow). The bronchus is lined with degenerated epithelial cells (white arrow) and exfoliated epithelial cells seen in the lumen (star). (**e**) Sitagliptin + BUS: showing more or less ordered lung construction with mild cellular invasion (arrowhead) and slightly thickened interalveolar septa (double-headed arrow). Bronchus appeared with few degenerated epithelial cells. Testis. (**f**) Control: showing the testis formed of numerous packed seminiferous tubules (T) containing spermatogenic cells at various stages; spermatogonia (Sg), primary spermatocytes (Ps), spermatids (Sp), and sertoli cells (red arrows) in between. The lumen contained spermatozoa (Sz). Narrow normal interstitial spaces (*) containing interstitial Leydig cells (arrowheads) are noticed between the tubules. (**g**) Sitagliptin: showing closely packed seminiferous tubules (T) lined by normal germinal epithelium (rectangle) and narrow interstitial spaces in between (*) (**h**) BUS: showing distorted (red arrow), detached (curved arrow) part of basement membrane and separated germinal epithelium (black arrow). Most germ cells are degenerated and have lost their morphologies (circle), with few spermatozoa within their lumen (Sz). (**i**) Most germinal epithelial cells appeared vacuolated (V), and others exhibit pyknotic nuclei (arrows). Wide intertubular spaces with uniform acidophilic material (*) and several vacuoles (arrowheads). Congested engorged blood vessels (BV) are also seen. (**j**) Sitagliptin + BUS: showing significantly improved architecture with almost normal organized tubules (T) with normal spermatogenesis; however, few cells appeared with pyknotic nuclei (black arrow) and vacuoles (red arrow) (**a,b,c,d,e** scale bar 20 μm, × 400; **f,g,h,i,j** scale bar 50 μm, × 200).

H&E sections of the lung of the control and sitagliptin groups revealed numerous alveoli, alveolar sacs, blood vessels, and bronchi lined with simple columnar epithelium. The alveoli were parted by delicate interalveolar septa and lined by two types of cells: type I and II pneumocytes (Fig. 5a,b). The BUS group exhibited disorganized lung architecture with marked inflammatory cellular infiltration, extravasation of RBCs, and thickening of interalveolar septa. The bronchus is lined with degenerated epithelial cells and exfoliated cells seen in the lumen (Fig. 5c,d). The sitagliptin + BUS group showed marked improvement of the lung structure with mild cell infiltrate and slight thickening of interalveolar septa. Bronchus appeared with a few degenerated epithelial cells (Fig. 5e).

Testicular tissue of the control and sitagliptin groups revealed bursting seminiferous tubules with germinal epithelium lining containing numerous cell types, including spermatogonia, primary spermatocytes, and spermatids. The lumen contained spermatozoa. Sertoli cells are essential in germ cell growth. Interstitial Leydig cells are found in a narrow space between the seminiferous tubules (Fig. 5f,g). Seminiferous tubules of the BUS group exhibited lost architecture, distortion of the basement membrane, and separated germinal epithelium. Most germ cells appeared to have degenerated and lost their morphology, with only a few spermatozoa within their lumen.

Moreover, most germinal epithelial cells appeared vacuolated, and others exhibited pyknotic nuclei. Homogenous acidophilic material with many vacuoles appeared in the intertubular space. Congested engorged blood vessels are also seen (Fig. 5h,i). Sitagliptin treatment showed significant improvement in histological architecture; however, a few cells appeared with pyknotic nuclei and others with vacuoles (Fig. 5j).

### The effect of sitagliptin on Masson’s trichrome stain

**Figure 6:**
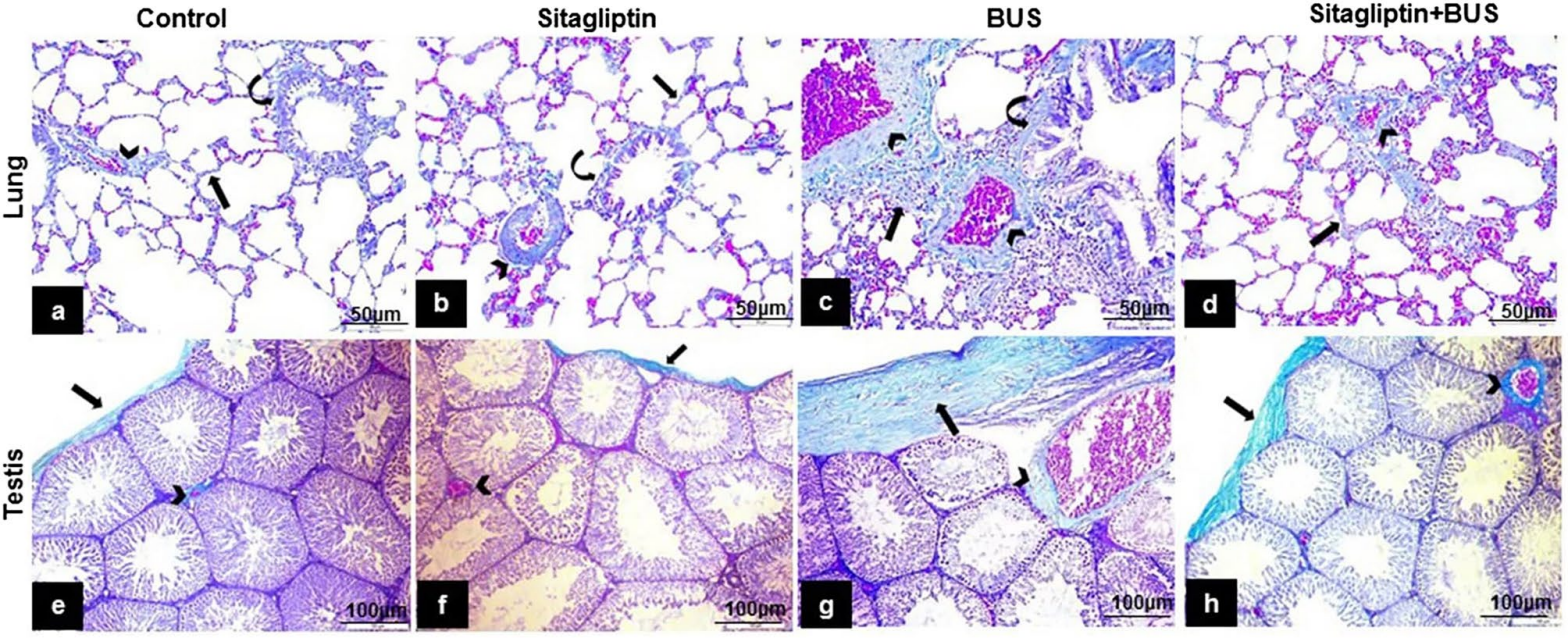
Masson’s trichrome staining of lung and testis tissues from different groups. *Lung*. (**a**) Control and (**b**) sitagliptin groups: showing minimal collagen fibers in interalveolar septa (arrow), around bronchus (curved arrow), and blood vessels (arrowhead). (**c**) BUS: showing dense collagen fiber buildup (arrows) in thickened interalveolar septa around the bronchus (curved arrow) and blood vessels (arrowheads). (**d**) Sitagliptin + BUS showing moderate collagen fibers deposition in interalveolar septa (arrow) and around the blood vessels (arrowhead). Testis. (**e**) Control and (**f**) sitagliptin groups: showing minimal collagen fibers deposition in the testicular capsule (tunica albuginea) (arrow) and around blood vessels (arrowhead). (**g**) BUS: showing dense collagen fibers deposition in tunica albuginea (arrow) and around blood vessels (arrowhead) (**h**) Sitagliptin + BUS showing moderate collagen fibers deposition in tunica albuginea (arrow) and around blood vessels (arrowhead) (**a,b,c,d** scale bar 50 μm, × 200; **e,f,g,h** scale bar 100 μm, × 100).

The control (Fig. 6a) and the sitagliptin (Fig. 6b) groups exhibited slight collagen fibers deposition in the lung. Compared to the control group, the BUS group revealed a marked accumulation of collagen in the lung (Fig. 6c). Sitagliptin treatment showed a notable decline in lung collagen deposition (Fig. 6d) compared to the BUS group. Regarding the testis, slight collagen fibers deposition was detected in control (Fig. 6e) and sitagliptin (Fig. 6f) groups. Compared to the control group, the BUS group revealed a marked accumulation of collagen (Fig. 6g). Sitagliptin-treated group showed attenuated testicular collagen deposition (Fig. 6h) compared to the BUS group.

### The effect of sitagliptin on area percentage of collagen deposition and area percentage of caspase-3 expression scoring in lung and testis

**Figure 7:**
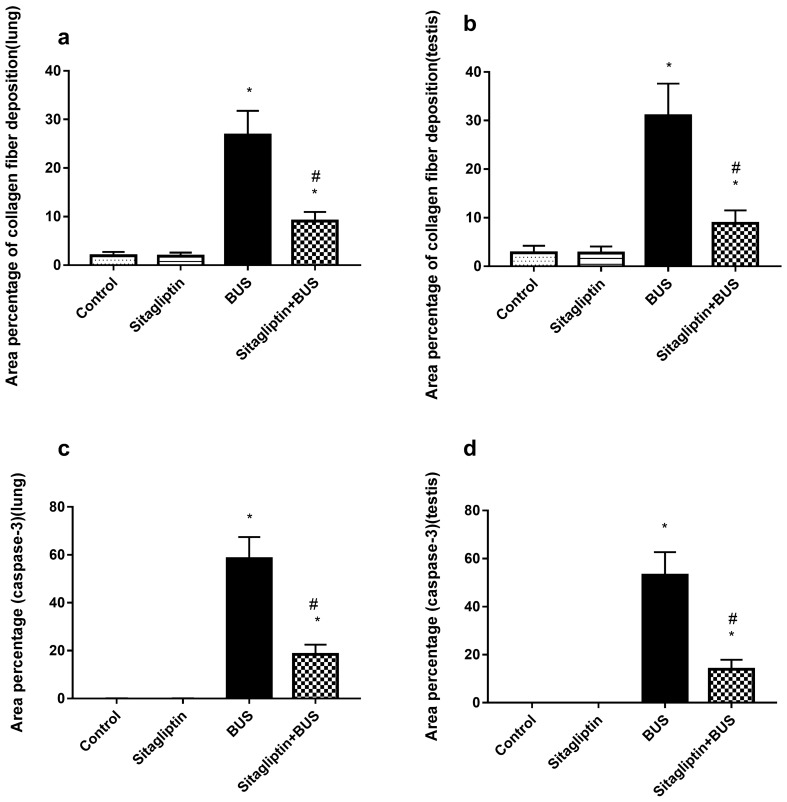
The effect of sitagliptin and BUS on area percentage of collagen deposition in (**a**) lung and (**b**) testis and area percentage of caspase-3 expression in (**c**) lung and (**d**) testis. Bars represent mean ± SD (n = 10). One-way ANOVA and the Tukey post hoc test were used to evaluate the data. *P < 0.05 compared to the control group; ^#^P < 0.05 compared to the BUS group.

As described in Fig. 7a, BUS significantly (*P* < 0*.*05) increased the area percentage of collagen deposition in the lung compared to the control group, while treatment with sitagliptin reduced BUS-induced increase in the area percentage of collagen deposition. Nevertheless, the area percentage of collagen deposition in the lungs of the sitagliptin-treated group was higher than that of the control group. Also, Fig. 7b illustrates that BUS increased the area percentage of collagen deposition in the testis compared to the control group (*P* < 0.05). Sitagliptin treatment reduced the area percentage of collagen deposition in the testis (*P* < 0*.*05) compared to the BUS group. However, the sitagliptin-treated group had more collagen deposition percentage area than the control group (*P* < 0*.*05).

In Fig. 7c, the administration of BUS significantly upregulated caspase-3 expression in the lung (*P* < 0.05) compared to the control group. Sitagliptin treatment reversed BUS-induced lung caspase-3 upregulation, but the control group's normal lung caspase-3 expression level was not obtained (*P* < 0.05). Likewise, testis caspase-3 expression was increased (*P* < 0.05) in the BUS group compared to that in the control group. Treatment with sitagliptin reduced (*P* < 0.05) testis caspase-3 expression compared to that in the BUS group. However, the testis caspase-3 expression level of the sitagliptin-treated group was higher (*P* < 0.05) than that of the control group level (Fig. 7d).

### The effect of sitagliptin on caspase-3 immunostaining

**Figure 8:**
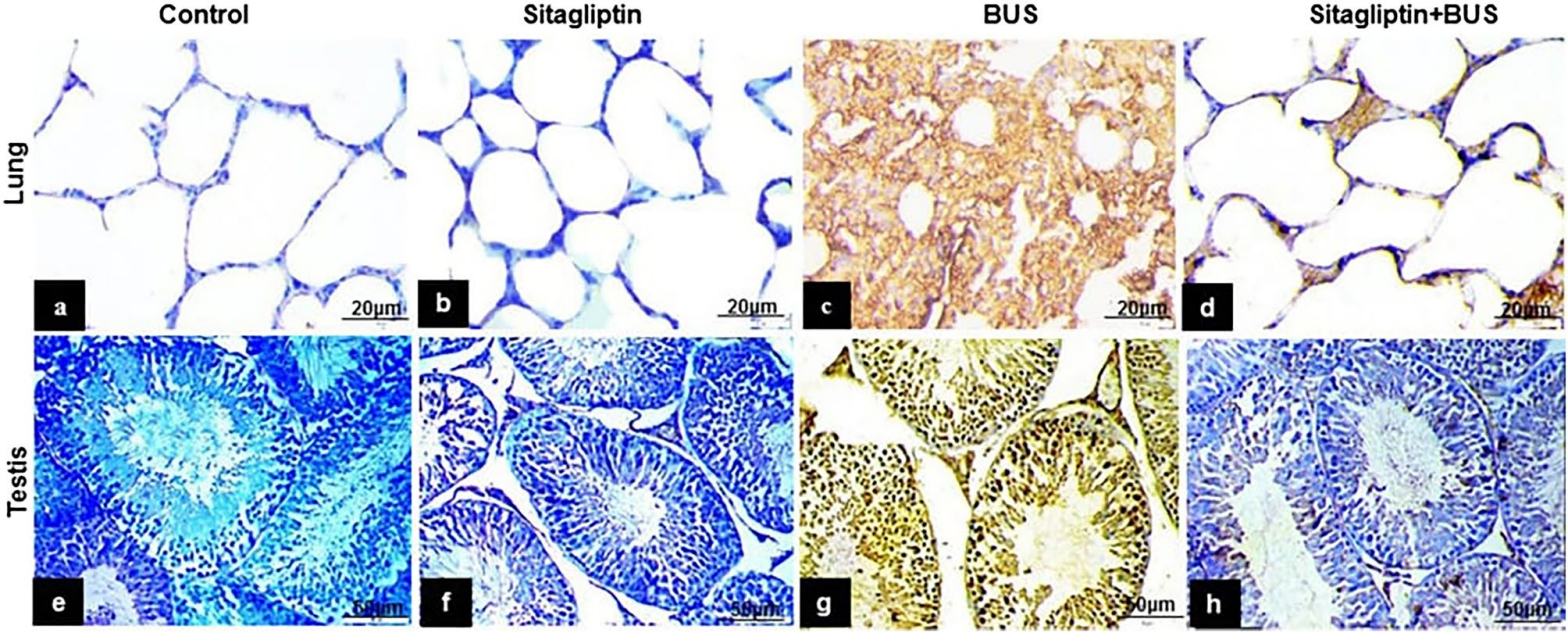
Caspase-3 staining of lung and testis tissues from various groups. Minimal to negative lung caspase-3 expression was demonstrated in the (**a**) control and (**b**) sitagliptin groups. (**c**) BUS dramatically upregulated the expression of caspase-3 in lung tissue. (**d**) Sitagliptin + BUS revered BUS-induced increase in lung caspase-3 expression. Regarding the testis, minimal to negative caspase-3 expression was detected in the (**e**) control and (**f**) sitagliptin groups. (**g**) BUS induced a notable increase in caspase-3 expression. (**h**) Sitagliptin + BUS significantly downregulated caspase-3 expression (**a,b,c,d** scale bar 20 μm, × 400; **e,f,g,h** scale bar 50 μm, × 200).

The control (Fig. 8a) and the sitagliptin (Fig. 8b) groups exhibited negative to minimal caspase-3 expression in the lung. Administration of BUS significantly upregulated caspase-3 expression in the lung (Fig. 8c) compared to the control group. Sitagliptin treatment induced a marked reduction in lung caspase-3 expression (Fig. 8d) compared to the BUS group. Similarly, negative to minimal testis caspase-3 expression was observed in the control (Fig. 8e) and sitagliptin (Fig. 8f) groups. BUS markedly upregulated caspase-3 expression in the testis (Fig. 8g) compared to the control group. Sitagliptin treatment induced a marked reduction in testis caspase-3 expression (Fig. 8h) compared to the BUS group.

## Discussion

Busulfan is an alkylating anti-cancer drug used on a wide scale for treating a variety of solid and hematological neoplasms. Despite the drug’s efficacy, its use is limited due to pulmonary and testicular toxicity^2,3^. We discovered that sitagliptin reversed pulmonary and testicular damage induced by BUS through pathways connected to the prevention of oxidative stress, the release of inflammatory mediators, and the presence of fibrotic and apoptotic components in the lung and testes of rats.

The current study noted a significant body weight loss in the BUS group. This result agreed with that of Hosseini Ahar et al.^40^, which concluded that weight loss could be due to the cytotoxic effect of BUS^40^. A contradictory study noted no difference in body weight change in BUS compared to the control group^41^. Treating BUS toxicity with sitagliptin significantly attenuated weight loss compared to the BUS group.

Pulmonary fibrosis has been linked to the production of reactive oxygen species resulting from oxidative stress^7^. This was confirmed in the current study when BUS significantly increased MDA levels in the lung; additionally, BUS decreased antioxidant capacity in the lung by significantly lowering GSH levels, resulting in a disrupted oxidant/antioxidant balance. This outcome was in line with the findings of Elhadidy et al.^7^. Sitagliptin treatment significantly restored oxidant/antioxidant balance by lowering lung MDA levels while increasing GSH levels. Previous studies have shown that sitagliptin has an antioxidant effect^19^.

BUS-induced DNA damage and initiation of oxidative stress through transferal of its alkyl group(s) to various body cells, including the lung, may explain the marked increase in lung index, indicating pulmonary fibrosis^7^. Sitagliptin administration significantly reduced lung index compared to the BUS group.

It has been noted that decreased expression of *SIRT1* was linked to BUS-induced oxidative stress^42^. Our study clarified that the downregulation of *SIRT1* expression in the lung of the BUS group significantly increased oxidative stress compared to the control group. Additionally, we found that the increase in serum TNF-α level was accompanied by downregulation of *SIRT1* in the BUS group compared to the control group. *SIRT1* upregulation is found to exert anti-inflammatory effects through inhibition of the production of inflammatory cytokines^11,43^.

Increased TNF-α level has been previously recorded in BUS-induced pulmonary and testicular injury studies^7,44^. In our study, serum analysis revealed that BUS significantly increased TNF-α levels. In contrast, treatment with sitagliptin markedly reduced serum TNF-α levels denoting its beneficial anti-inflammatory effect. The anti-inflammatory effect of sitagliptin has been observed previously^16,45^ and could be due to the upregulation of the *SIRT1* gene^46^*.*

In the present study, BUS-induced lung inflammation was ascertained by the histopathological picture showing loss of normal lung architecture with inflammatory cell infiltration and thickening of interalveolar septa. This finding agreed with that of Elhadidy et al.^7^. Sitagliptin treatment improved the histopathological picture of the lung tissue compared to the BUS group. Sitagliptin showed similar findings in a pulmonary injury animal model by downregulating the proinflammatory cytokines, including TNF-α^16^*.*

The present study showed that BUS increased collagen deposition in the lung compared to the control group. BUS-induced oxidative stress and inflammation stimulated the lung fibroblasts, consequently increasing collagen synthesis and disrupting the balance between collagen deposition and reabsorption^7^. Other postulated mechanisms of fibrosis include downregulation of *SIRT1* and apoptosis^11,47,48^. Sitagliptin treatment attenuated BUS-induced deposition of collagen in the lung. The antifibrotic effect of sitagliptin has been reported before^20^*.*

Increased *FOXO1* expression has been linked to apoptosis^10^. The current study detected a marked increase in *FOXO1* expression in the BUS group compared to the control group indicating apoptosis. *SIRT1*-induced inhibition of *FOXO1* by deacetylation is attributed to inhibiting apoptosis^12^*.* Accordingly, the current study revealed that treatment with sitagliptin ameliorated BUS-induced increase in lung *FOXO1* expression due to the upregulation of lung *SIRT1* expression.

As shown in the current study, apoptosis was confirmed by increased expression of caspase-3 in the lung tissue in the BUS group compared to the control group. This finding was also reported previously^7^. Treatment with sitagliptin contributed to a noticeable reduction in lung caspase-3 expression. The antiapoptotic effect of sitagliptin was demonstrated by previous studies^19,45^*.*

BUS is a chemotherapeutic agent that affects rapidly dividing cells like the testis leading to testicular toxicity and sterility^5^. Oxidative stress impairs reproductive function and eventually leads to infertility^44,49^. The current study noted that BUS increased testicular MDA levels and decreased the antioxidant testicular levels of GSH. This finding agreed with that of Abarikwu et al.^44^.

It is thought that the number of germ cells determines the testicular weight. As a result, reduced testicular weight is considered a good indicator of testicular toxicity that could be caused by oxidative stress and reduction of testosterone production^22,50^. Moreover, BUS leads to a considerable reduction of sperm count due to a cessation of spermatogonia differentiation^22^. Accordingly, the current study demonstrated that BUS markedly lowered the testis index compared to the control group, which was also documented by Abarikwu et al.^51^ and Hosseini Ahar et al.^40^. Conversely, Hakemi et al.^41^ observed no difference in testis index between the BUS and control groups. Sitagliptin treatment attenuated BUS-induced oxidative injury by elevating MDA and lowering GSH testicular levels, which, in turn, was reflected in the testicular index and sperm count, denoting amelioration of germ cell loss by BUS. Sitagliptin was reported to have antioxidant properties on the testis^45^.

Also, in the current study, sperm viability and motility were decreased after BUS injection, while abnormal morphology showed a significant increase. Motility reduction could be explained by BUS-induced oxidative stress that affects the fluidity of the tail membrane of the sperm cell due to disturbing the polyunsaturated fatty acids it contains. Also, BUS leads to sperm flagella length reduction^41^. In contrast, abnormal sperm morphology could be attributed to BUS-induced impairment of spermatogenesis, the fertilizing ability of sperms, and germ cell adherence to seminiferous epithelium^52,53^. These findings were consistent with Vafaei et al.^52^ and Abd-Elrazek and Ahmed-Farid^34^. Sitagliptin treatment exhibited a significant increase in sperm motility and viability and reduced abnormal morphology due to its antioxidant effect^17^. In agreement, sitagliptin improved sperm parameters in other models of testicular toxicity^17,45^.

Testosterone is essential for sexual function and fertility^54^. Besides oxidative stress, BUS-induced reduction in testosterone levels could be attributed to inflammation and apoptosis^41,44^. In the current study, BUS injection markedly reduced testosterone levels. Other studies^52,55^ have also reported this finding. Sitagliptin-treated group significantly increased testosterone levels compared to the BUS group. Previous models of testicular injury have shown that sitagliptin influenced testosterone levels^17,45^.

As mentioned, *SIRT1* controls various pathways, including oxidative stress, inflammation, fibrosis, apoptosis, and autophagy. *FOXO1* is one of the proteins regulated by *SIRT1*^11^. The current study detected that testicular *SIRT1*/*FOXO1* balance was disrupted by BUS, and this balance was restored by sitagliptin treatment. Hence, the current study highlighted that modulation of *SIRT1/FOXO1* interaction by sitagliptin could attenuate oxidative stress, inflammation, fibrosis, and apoptosis. This study demonstrated that BUS increased testicular collagen deposition that was attenuated by sitagliptin treatment.

The present study demonstrated that BUS increased TNF-α levels and resulted in distorted testicular architecture and degeneration of germ cells. Germinal epithelial cells appeared vacuolated with pyknotic nuclei. These findings agreed with the study of Fotouh et al.^48^. Treatment with sitagliptin improved BUS-induced histopathological changes in the testicular tissues.

BUS causes oxidative stress and the generation of reactive oxygen species (ROS). ROS causes impairment of the mitochondrial membrane leading to activation of caspase-3 expression, indicating apoptosis^8^. Also, increased *FOXO1* expression leads to apoptosis^10^. In agreement, the current study showed that BUS increased the expression of caspase-3 in the testis by increasing oxidative stress and *FOXO1* expression. This finding was also observed by previous studies^8,56^. Caspase-3 expression in the testis was decreased by sitagliptin treatment. The antiapoptotic effect of sitagliptin on the testis was demonstrated previously^45^*.*

To conclude, sitagliptin exhibited protective effects against BUS-induced pulmonary and testicular toxicity. Sitagliptin-induced amelioration of oxidative stress, inflammatory, apoptotic, and fibrotic markers might be responsible for the protective effect of DPP4Is on pulmonary and testicular tissues. Thus, sitagliptin in the investigated dose could hold promise for preventing pulmonary and testicular toxicity in patients receiving BUS.

## Data Availability

The data used in the current study are available upon request from authors.
